# Abscess of the clivus in an adolescent with complicated acute rhinosinusitis: a case report

**DOI:** 10.1186/s13052-020-00863-y

**Published:** 2020-07-14

**Authors:** Lorenzo Solimeno, Sara Torretta, Paola Marchisio, Elisabetta Iofrida, Samantha Bosis, Claudia Tagliabue, Giada Maria Di Pietro, Lorenzo Pignataro, Claudio Guastella

**Affiliations:** 1 Fondazione IRCCS Ca’ Granda Ospedale Maggiore Policlinico, Via F. Sforza 35,20122, Milan, Italy; 2 Department of Clinical Sciences and Community Health, University of Milan, Milan, Italy; 3 Department of Pathophysiology and Transplantation, University of Milan, Milan, Italy

**Keywords:** Orbital abscess, Sinusitis, Children, Clival abscess, Case report

## Abstract

**Background:**

Complications of acute sinusitis affecting multiple sites are very uncommon, and generally develop for a delayed diagnosis of the primary infection, with possible severe and life-threatening evolution. Patients can have variable presentations according to the site and extent of the infection. Multiple forms generally include the coexistence of orbital manifestations and intracranial infections. We here present a case with unusual multiple sites locations (i.e.: intraorbital intraconic abscess, sigmoid sinus thrombosis, preclival abscess, multiple splanchnocranium osteomyelitic processes).

**Case presentation:**

A 13-year-old male presented at our hospital with right progressive orbital oedema with eyesight worsening and signs of meningitis. Computed tomography and magnetic resonance (MRI) demonstrated right intraorbital intraconic abscess, left sphenoidal sinusitis, transverse and sigmoid sinus thrombosis. Ophthalmologic evaluation documented a right optic nerve sufferance. Endoscopic and superior right trans-palpebral surgical decompression was performed, and the abscess was drained. Microbiological analysis revealed the presence of multi-sensitive Streptococcus Intermedius. Subsequent prolonged antibiotic and anti-thrombotic treatments were started. In the following two-weeks the sinusal and ophthalmologic clinical conditions improved, whereas the patients complained of mild to moderate cervical pain and suffered from intermittent pyrexia. Control MRI documented clival abscess extending up to preclival soft tissues posterior to the nasopharynx, associated with mandible osteomyelitis, occipital condyles and anterior part of the temporal bone hyper intensity. Endoscopic trans-nasal surgical approach to the clival compartment with neurosurgery navigation-guided achieved preclival abscess drainage. Complete clinical and radiological recovery was achieved after 45 days of medical treatment.

**Conclusions:**

Multiple sites complicated rhinosinusitis is uncommon, and its management is challenging. A proper history and thorough clinical examination along with a radiological evaluation are key factors in the final diagnosis of patients with complicated multiple sites acute rhinosinusitis. A quick multidisciplinary approach is always necessary to avoid unwanted life-threatening complications.

## Background

Sinonasal-related orbital infections (SROIs) are typically paediatric diseases that occur in about.

3–4% of children with acute rhinosinusitis, and mainly affect children aged less than 5 years. They are generally due to ethmoidal sinusitis spreading to the orbit and are more frequent during winter [1].

SROIs are characterised by various clinical manifestations that may develop anteriorly or posteriorly to the orbital septum, a thin fibrous membrane extending from the orbital rims to the eyelids (the anterior boundary of the orbit) that acts as a barrier against the spread of external infections to the deep orbit. Chandler’s classification [2] distinguishes pre-septal complications, such as pre-septal cellulitis (POC, Chandler stage 1), which affects the eyelids and adnexa, without extending beyond the peri-orbit, from the more dangerous infections that develop posteriorly to the orbital septum, i.e., orbital cellulitis (OC, Chandler stage 2), sub-periosteal abscess (SPA, the collection of pus in the lamina papyracea, Chandler stage 3), or orbital abscess (OA, Chandler stage 4) [2]. Chandler stage 5 disease refers to cavernous sinus thrombosis [2]. Posterior septal complications are particularly dangerous, as they may lead to visual loss and life-threatening events, such as an intracranial abscess and cavernous sinus thrombosis. SPA accounts for 9–28% of all SROIs [3] and is generally secondary to ethmoiditis; it is more frequently located in the medial portion of the orbit [3], but other less frequent locations include superior and superomedial SPA generally due to frontal sinusitis [3]. Some authors have suggested that osteitis of the lamina papyracea may be one of the causative factors [3, 4].

The clinical presentation of SROIs includes swelling and redness around the eye, and possibly a temperature and impaired general condition, which are generally more frequent in the case of deep orbital involvement. The clinical signs suggesting post-septal complications due to increased intra-orbital pressure are proptosis, chemosis, ophthalmoplegia, diplopia, impaired visual acuity, or impaired red-green visual perception [5]. Although proptosis and ophthalmoplegia are strong predictors of advanced disease [6], computed tomography (CT) findings cannot differentiate OC from OA and may be absent in some patients with intra-orbital involvement [6]. It has been reported that proptosis and ophthalmoplegia may be absent in up to 50% of patients with Chandler stage≥3 [7], and so some authors have suggested that, in addition to a clinical examination, consideration should be given to other predictors, such as a high neutrophil count, older age, and a body temperature of > 39 °C.

SROIs are ophthalmic emergencies that need to be promptly recognised and treated in an urgent-care setting because of the possible risk of permanent visual loss due to optic neuritis or orbital nerve ischemia following orbital compression or stretching secondary to sub-periosteal or intra-orbital collections or orbital vein thrombosis. Life-threatening sequelae, such as cavernous sinus thrombosis, meningitis, and epidural or sub-dural abscess, may also occur as a result of intracranial spreading [8–10].

Multiple forms generally include the coexistence of orbital manifestations and intracranial infections. We here describe an unusual case of complicated acute rhinosinusitis presenting with multiple site infections (i.e.: intraorbital intraconic abscess, sigmoid sinus thrombosis, preclival abscess, multiple splanchnocranium osteomyelitic processes) in a previously healthy adolescent.

## Case presentation

**Fig. 1:**
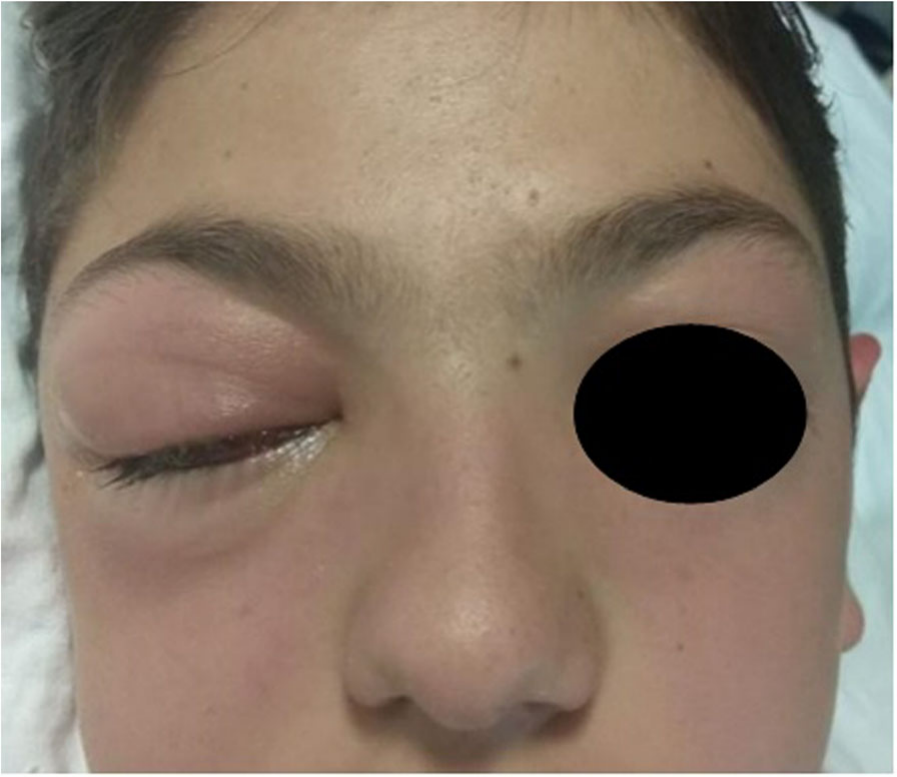
Clinical presentation of orbital cellulitis with swelling and hyperemia of the right eyelids (upper more than inferior)

A previously healthy, fully immunized 13 years-old boy was transferred to the intensive care unit department of our hospital with drowsiness, progressing painful hyperaemic right periorbital swelling, fever, bilateral nasal obstruction (right > left) and right purulent rhinorrhoea (Fig. 1). Moreover, he presented worsening right visual acuity, right dyschromatopsia and a dull cervical pain for 2 days. His mother mentioned us about a right orbital trauma during a sport event (soccer game) occurred 7 days before.

During ENT evaluation anterior rhinoscopy was performed with the finding of scarce purulent discharge from the right nasal cavity, with generalized hyperaemia and swelling of the nasal mucosae. Nasal fiberoptic endoscopy was difficult to perform due to generalized swelling of the nasal mucosae and only revealed right nasopharyngeal purulent drip. Respiratory space revealed normal.

On admission blood tests documented leucocytosis with neutrophilia and elevated C-reactive protein (CRP).

**Fig. 2:**
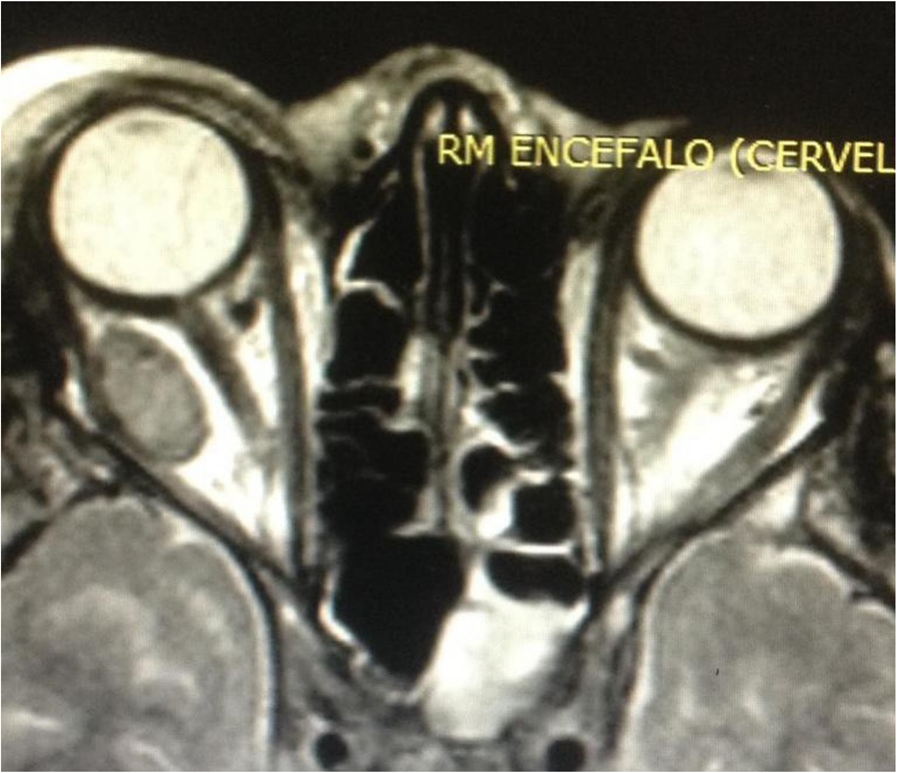
Preoperative MR imaging (axial view): right intraorbital intraconic abscess with signs of left sphenoiditis

**Fig. 3:**
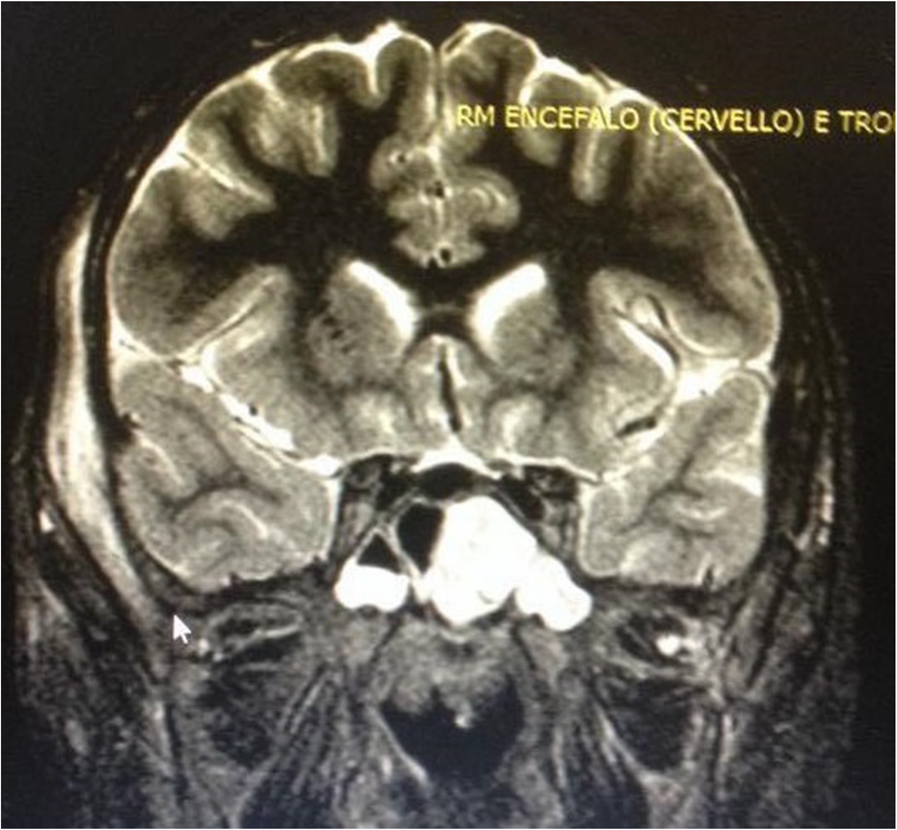
Preoperative MR imaging (coronal view): signs of bilateral sphenoiditis (left more than right)

**Fig. 4:**
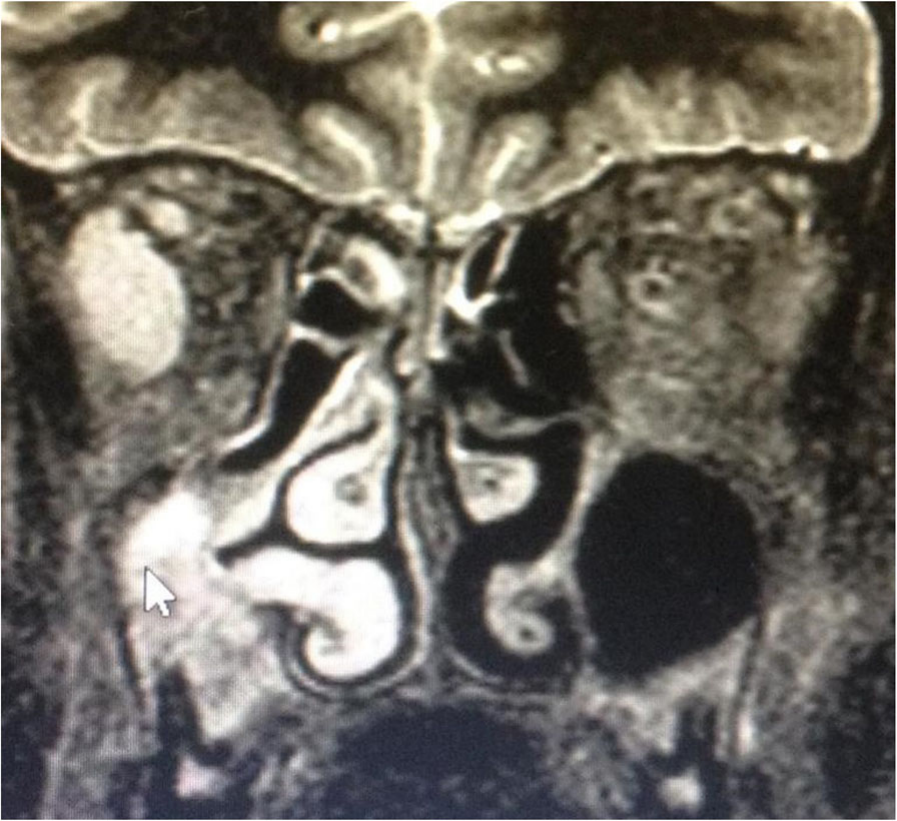
Preoperative MR imaging (coronal view): right intraorbital intraconic abscess and signs of acute maxillary sinusitis

A brain and maxillo-facial contrast-enhanced computed tomography (CT) and magnetic resonance imaging (MRI) were performed and revealed the presence of a right intraorbital intraconic abscessual formation (Figs. 2, 3, 4), acute right maxillary and left sphenoid sinusitis, minimal clival bone erosion and signs of sigmoid and transverse venous sinuses thrombosis.

Urgent ophthalmologic evaluation was requested and resulted difficult to be performed due to the minimal palpebral fissure; however, it demonstrated signs of right optic nerve sufferance.

Based on this finding, a large spectrum intravenous antibiotic treatment with ceftriaxone (2 g twice a day) and metronidazole (500 mg four times a day) and an anticoagulant treatment with subcutaneous low-molecular-weight heparin were introduced.

The boy underwent an urgent surgical treatment with drainage of the right intraorbital intraconic abscess via right superior trans-palpebral approach and right antrostomy and left sphenoidotomy via endoscopic sinus surgery (ESS). Microbiological tests of the purulent collection were positive for multisensible Streptococcus intermedius.

Full recovery of both orbital swelling and nasal complaints was achieved few days after surgery, as well as a progressive improvement with complete recovery in visual acuity.

Antibiotic treatment with ceftriaxone and metronidazole was continued for 2 weeks, when worsening signs of meningeal sufferance (torcicollis and rigor nucalis), bradycardia and intermittent fever occurred.

Lumbar puncture, abdominal and cardiac ultrasound (US) and blood culture were performed, but all revealed negative for meningitis, further abscessual formations and bacteremia. Immunological tests including immunoglobulins and IgG subclassis, lymphocyte subpopulations, tests for complement function were performed and resulted negative.

**Fig. 5:**
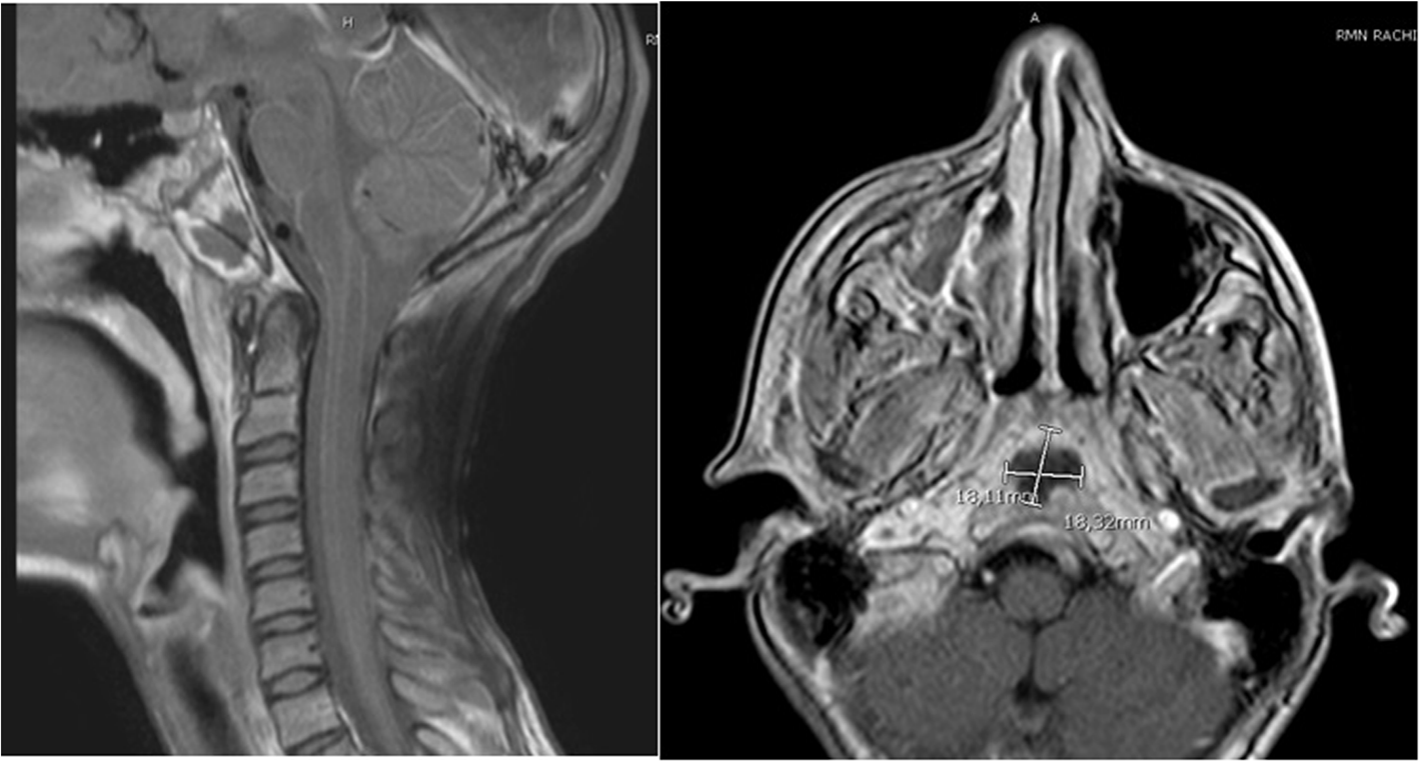
Axial and sagittal MR images of abscess of the clivus

Control head MRI was performed and documented the presence of an abscessual collection (Fig. 5) of the preclival region extending to the preclival soft tissue posteriorly to the nasopharynx and to the cervical long muscles’ insertion, associated with mandibular, occipital condyles and anterior part of the occipital bone osteomyelitis.

Multidisciplinary team discussion with neurosurgeon, infectivologist, paediatricians and radiologist was achieved and another surgical urgent procedure was performed: partial posterior septectomy, bilateral sphenoidotomy with access to the preclival compartment and preclival abscess drainage in ESS.

Antibiotic treatment was changed to vancomycin (40 md\kg\die in three doses) and meropenem (100 mg\kg\die in three doses) and continued for 34 days with fully complete recovery of the infection and discharged of the patient after ENT, ophthalmologic, neurologic and hematologic controls were referred as negative. Semi-annual imaging with contrasted MRI, as well as ENT, ophthalmologic and neurologist outpatient clinic controls were performed. No recurrences have been detected during the follow-up period (16 months).

## Discussion and conclusions

This clinical case is very intriguing because, as we know from the recent history of this patient, he had been suffering from some form of nasal infection in the days before the development of orbital cellulitis (developing into an abscess), plus he coped with a traumatic event during a soccer game 7 days before the development of the symptoms. In fact, we can recognise in this case two mechanisms possibly acting as predisposing factors for brain abscess: the direct extension stemming from the ENT districts (mastoid, middle ear or paranasal sinuses) and a penetrating head injury.

Multiple sites infections complicating acute rhinosinusitis in children are uncommon events: the presence of any SROI located posteriorly to the orbital septum predisposes the development of life-threatening intracranial complications [1, 8, 9, 11]. Literature documents that multiple forms generally include the coexistence of orbital manifestations and intracranial infections (meningitis, brain abscess, subdural empyema, cavernous sinus thrombosis) or inflammatory processes of the paranasal sinuses’ bony boundaries, such as frontal and maxillary osteomyelitis [11].

However, in this case secondary infectious focuses did not involve these sites, rather affected the preclival soft tissues posteriorly to the nasopharynx and more distant multiple bony subsites of the splanchnocranium (i.e.: mandible, occipital condyles, anterior temporal bone) were osteomyelitic processes were documented.

In this case it seems that the involved bacterium, *S. intermedius*, is the cornerstone. As a fact, *S. intermedius*, a commensal organism, has the potential to cause significant morbidity. *S. intermedius* expresses one or more members of a family of structurally and antigenically related surface proteins termed antigen I/II, which plays a potential role in its pathogenesis. It is involved in binding to human fibronectin and laminin and in inducing IL-8 release from monocytes, which promotes neutrophil chemotaxis and activation [12].

*S. intermedius*, together with *S. constellatus* and *S. anginosus*, is a member of the “*Streptococcus anginosus* group”. This group of bacteria is also frequently encountered in invasive suppurative infections at a range of sites, including liver and brain abscesses, dentoalveolar infections, and infective endocarditis.

The clinical association of *S. intermedius* with the tendency to form abscesses has long been recognized. The first case of brain abscess caused by *S. intermedius* was reported in 1975, where it was found to comprise a population of approximately 5.4% among other pathogens in 71 analysed brain abscess samples [12]. *S. intermedius* has been isolated from 50 to 80% of brain abscesses [12]. Several recent studies have implicated *S. intermedius* infection in the pathogenesis of brain abscess [12]. In 2006, a study reported the role of *S. intermedius* in cases of intracerebral abscess in children, resulting from haematogenous spread from a distant focus (e.g., congenital heart disease) or extension from a contiguous focus of infection (e.g., sinus, teeth, and middle ear) [12]. In this study, among children who developed brain abscess associated to *S. intermedius*, 58% of 17 patients had a cyanotic congenital heart disease, while 42% of patients had rhinosinusitis, otitis media, or dental caries [12].

Its tendency to form abscess should worry above all in patients with unexplained fever and other signs of organ involvement. In our case, for example, the patient presented intermittent fever, bradycardia and neck pain after the first surgical procedure (orbital abscess drainage): cerebrospinal fluid examination (resulted negative), trans-thoracic Ultrasound (TT-US, resulted negative) and brain CT were promptly requested and clival abscess was found to be the cause.

*S. intermedius* appears to be a major agent of brain abscess, as this bacterium possesses many virulence factors coupled with the recent emergence of its antibiotic-resistant strains. Therefore, understanding the roles of both host antibacterial immune responses along with its virulence factors may lead to the establishment of novel therapeutic treatments for brain abscess [12, 13].

Orbital cellulitis and abscess are severe diseases and prompt treatment is needed to avoid visual loss or intracranial complications. In these cases, an early diagnosis of a complication is important to avoid a much more dangerous and complex course. The clinical examination alone is not always diriment and therefore a CT scan can be useful to estimate the extent of the infection inside the orbit, while MRI can indicate intracranial spread. Initially, intra-venous antibiotics should be administered, but if no improvement appears within 48 h, surgical drainage of the orbit and the affected sinuses must be performed. In medial or medial-inferior abscess, a transnasal approach is possible, but in superior orbital abscess an external incision is required.

The clinical course, physical examination, and imaging findings of patients with SROI should all be used to determine the best treatment plan. If the patients have clinical worsening in their symptoms or examination while undergoing antibiotic therapy, few would argue against surgical therapy. However, the patient’s initial presentation can help in this decision making, as well.

## Data Availability

Not applicable.

## Abbreviations

CT: Computed tomography
MRI: Magnetic resonance imaging
SROI: Sinonasal-related orbital infection
CRP: C-reactive protein
POC: Pre-septal orbital cellulitis
OC: Orbital cellulitis
SPA: Sub-periosteal abscess
OA: Orbital abscess
ESS: Endoscopic sinus surgery
US: Ultrasound
TT-US: Trans-toracic ultrasound

## References

[1] Torretta S, Guastella C, Marchisio P, Marom T, Bosis S, Ibba T (2019). Sinonasal-related orbital infections in children: a clinical and therapeutic overview. J Clin Med.

[2] Buchanan MA, Muen W, Heinz P (2012). Management of periorbital and orbital cellulitis. Paediatr Child Health.

[3] Migirov L, Yakirevitch A, Bedrin L, Wolf M (2009). Endoscopic sinus surgery for medial orbital subperiosteal abscess in children. J Otolaryngol Head Neck Surg.

[4] Eviatar E, Sandbank E, Kleid J, Gavriel SH (2014). The role of osteitis of the lamina papyracea in the formation of subperiosteal orbital abscess in young children. Int J Pediatr Otorhinolaryngol.

[5] Fokkens WJ, Lund VJ, Mullol J, Bachert C, Alobid I, Baroody F (2012). EPOS 2012, European position paper on rhinosinusitis and nasal polyps 2012. A summary for otorhinolaryngologists. Rhinology.

[6] Jabarin B, Eviatar E, Israel O, Marom T, Gavriel H (2018). Indicators for imaging in periorbital cellulitis secondary to rhinosinusitis. Eur Arch Otorhinolaryngol.

[7] Rudloe TF, Harper MB, Prabhu SP, Rahbar R, Vanderveen D, Kimia AA (2010). Acute periorbital infections: who needs emergent imaging?. Pediatrics..

[8] Bedwell J, Bauman NM (2011). Management of pediatric orbital cellulitis and abscess. Curr Opin Otolaryngol Head Neck Surg.

[9] Kinis V, Ozbay M, Bakir S, Yorgancilar E, Gun R, Akdag M (2013). Management of orbital complications of sinusitis in pediatric patients. J Craniofac Surg.

[10] Sharma A, Liu ES, Le TD, Adatia FA, Buncic JR, Blaser S (2015). Pediatric orbital cellulitis in the Haemophilus influenzae vaccine era. J AAPOS.

[11] Ali A, Kurien M, Mathews SS, Mathew J (2005). Complications of acute infective rhinosinusitis: experience from a developing country. Singapore Med J.

[12] Mishra AK, Fournier PE (2013). The role of Streptococcus intermedius in brain abscess. Eur J Clin Microbiol Infect Dis.

[13] Prigitano A, Romanò L, Auxilia F, Castaldi S, Tortorano AM (2018). Antibiotic resistance: Italian awareness survey 2016. J Infect Public Health.

